# The Chick Embryo Chorioallantoic Membrane Assay as a Short-Term Exploratory Model for Cervical Cancer Research

**DOI:** 10.3390/life16010135

**Published:** 2026-01-15

**Authors:** Carlos César Patiño-Morales, Ricardo Jaime-Cruz, Raquel González-Pérez, Laura Villavicencio-Guzmán, Tania Cristina Ramírez-Fuentes, Marcela Salazar-García

**Affiliations:** 1 Developmental Biology Research Laboratory, Hospital Infantil de México Federico Gómez, Mexico City 06720, Mexico; cpatino@cua.uam.mx (C.C.P.-M.); ricardo.jaime.cruz@gmail.com (R.J.-C.); coiledstar@gmail.com (R.G.-P.); lauvillavi@gmail.com (L.V.-G.); tania.ramirez3190@outlook.com (T.C.R.-F.); 2 Cell Biology Laboratory, Universidad Autónoma Metropolitana-Cuajimalpa, Mexico City 05348, Mexico; 3 Department of Health Sciences, Universidad Tecnológica de México, UNITEC México-Campus Sur, Mexico City 09810, Mexico; 4 Facultad de Medicina, Universidad Nacional Autónoma de México, Mexico City 04360, Mexico; 5 Section of Graduate Studies and Research, School of Medicine, National Polytechnic Institute, Mexico City 11340, Mexico

**Keywords:** cancer, CAM-assay, cervical cancer, tumor development, *in ovo*, 3Rs

## Abstract

Cervical cancer (CC) remains a significant public health problem. Despite the availability of standard treatment strategies, chemotherapy-resistant tumors persist, highlighting the need to explore new therapeutic approaches or adjuvant strategies. This underscores the importance of preclinical in vivo models. Conventional models, such as murine xenografts, patient-derived xenografts (PDXs), and patient-derived organoids (PDOs), provide valuable biological relevance but are often time-consuming, costly, and resource-intensive. In this context, the chick embryo chorioallantoic membrane (CAM) assay represents a rapid, low-cost, and technically accessible in vivo platform. The CAM is a non-innervated, highly vascularized extraembryonic structure that provides a suitable environment for tumor generation from xenografts. However, despite the broad use of the CAM assay for tumor xenografts, standardized and comparative methodological optimizations specifically addressing technical variables for cervical cancer tumor induction remain limited. Therefore, the aim of this study was to optimize the CAM assay for tumor generation using the HeLa and SiHa cell lines. The generated tumors are vascularized and exhibit Ki-67 expression. The CAM assay is an excellent short-term exploratory model based on developing chicken embryos for studying the developmental biology of cervical tumors, which would accelerate the preclinical investigation of new therapeutic molecules.

## 1. Introduction

Cervical cancer (CC) remains a significant global public health problem, particularly in developing countries, with approximately 660,000 new cases and 350,000 deaths worldwide in 2022 alone. Human papillomavirus (HPV) types 16 and 18 are high-risk viruses responsible for more than 70% of CC cases worldwide [1,2,3]. Despite CC screening programs and preventive HPV vaccination, the incidence of this disease remains high, particularly in developing regions [4]. Recent advances in cancer therapy have shown positive preclinical and clinical results. However, many women exhibit resistance to therapy [5,6]. Thus, mortality from CC remains a significant concern, especially in advanced stages. Therefore, it is crucial to continue to develop preclinical models that can help identify new and improved therapies or therapeutic adjuvants to improve current treatments and allow their discovery in the context of personalized medicine. In this context, 2D cell cultures have been a valuable tool in CC research. Primary cell cultures and established cell lines have been developed from multiple tissues of the female reproductive tract [7,8,9,10,11]. Most in vitro research on CC has used cell lines such as HeLa, CaSki, SiHa, or C33-A [12,13,14]. Three-dimensional (3D) tumor models have emerged as a significant tool in preclinical cancer research due to their ability to better replicate the tumor tissue architecture compared to two-dimensional (2D) cell cultures [15,16]. Nonetheless, there is still a need for models that more accurately reflect the tumor microenvironment in patients, facilitating the study of processes such as angiogenesis and metastasis. Animal models have been extensively employed for this purpose, including the immunodeficient mice NOD-SCID and nude mice [17,18]. Additionally, K14-E7/ΔN87βcat mice, which are known to develop invasive cervical squamous carcinomas within a six-month period, have also been used [19]. Moreover, the zebrafish model has also been used for this purpose [20]. Although the aforementioned models have proven valuable for tumor establishment in CC research, they come with some disadvantages: tumor formation in mice models typically requires several weeks. It also needs a special animal facility and involves high costs and maintenance. Although murine and zebrafish models provide robust and highly standardized platforms for tumor biology studies, they require specialized animal facilities and longer experimental planning. The CAM assay represents a complementary in vivo model that enables short-term tumor growth, direct visualization, and localized manipulation within a simplified regulatory framework. When appropriately optimized, it may facilitate parallel testing of experimental conditions, particularly during early-stage exploratory studies [21,22]. The chicken embryo CAM assay serves as a short-term exploratory model that is naturally immunosuppressed up to day 14 of development. It provides a flexible, cost-effective, and less ethically controversial option for studying tumor developmental biology [23,24]. The CAM structure fuses the allantois and the chorion and is formed by days 3.5–4 and appears fully differentiated between days 9–12. It has a rich vascular network of arteries and veins, which makes it ideal for the maintenance of xenotransplants, as it provides tumor cells with the essential nutrients required for growth [25]. Tumor-secreted angiogenic factors allow for the ingrowth of proliferative host vessels that supply oxygen to the graft. Although there is an initial avascular phase, neovascularization of the graft begins approximately 72–96 h after implantation [26]. The model is widely used as an in vivo system to study various types of tumors [27] and processes such as angiogenesis and metastasis [28,29]. In this study, tumors were induced from a cell suspension in Matrigel using the HeLa and SiHa cell lines, demonstrating that the (*in ovo*) CAM assay can be used to induce tumors. This model represents a very practical short-term exploratory model for CC research, especially for laboratories that do not have access to a bioterium.

## 2. Materials and Methods

### 2.1. Cell Culture

HeLa and SiHa cell lines were obtained from the American Type Culture Collection (ATCC) and cultivated in DMEM/F12 (Gibco, Grand Island, NY, USA) supplemented with 10% fetal bovine serum (Gibco, Grand Island, NY, USA). All the cell lines were cultivated in T75 flasks coated with 2% gelatin at 37 °C in a 5% CO_2_ incubator. All cultures were incubated until reaching 70% confluency. The culture medium was removed from the flasks to harvest the cells. The cells were washed two times with PBS and then 500 μL of 0.25% trypsin (Gibco, Grand Island, NY, USA) and 500 μL of versene (PBS with 0.04% EDTA) were added. Once the cells detached from the plate, 2 mL of the medium supplement was added to inactivate the trypsin. The cells were collected in a Falcon 15 mL tube and centrifuged at 800 rpm to collect the cell pellet, which was washed twice with PBS. Tumor induction onto the chick embryo chorioallantoic membrane.

### 2.2. Tumor Induction onto the Chick Embryo Chorioallantoic Membrane

Fertilized White Leghorn chicken eggs from a local poultry farm (ALPES, Puebla, Mexico) were used and incubated at 37 °C with 60% relative humidity in an incubator with constant movement until the experiments were conducted. A total of 360 fertilized chicken eggs were used per experimental condition (A–D), corresponding to 180 eggs inoculated with HeLa cells and 180 eggs inoculated with SiHa cells. Each experimental variable was evaluated using a separate set of eggs. For each variable, eggs were equally distributed into two experimental groups as follows: (A) Days of incubation prior to tumor induction: Group 1, cell placement onto the CAM on day 4 of incubation (n = 90 HeLa and n = 90 SiHa); Group 2, cell placement onto the CAM on day 7 of incubation (n = 90 HeLa and n = 90 SiHa). (B) CAM laceration: Group 1, without CAM laceration (n = 90 HeLa and n = 90 SiHa); Group 2, with CAM laceration (n = 90 HeLa and n = 90 SiHa). (C) Use of Matrigel (Corning, Corning, NY, USA, Cat. #354234): Group 1, cell inoculation without Matrigel (n = 90 HeLa and n = 90 SiHa); Group 2, cell inoculation with Matrigel (n = 90 HeLa and n = 90 SiHa). (D) Use of trypsin: Group 1, cell inoculation without trypsin treatment (n = 90 HeLa and n = 90 SiHa); Group 2, cell inoculation with trypsin treatment (n = 90 HeLa and n = 90 SiHa). 3 × 10^6^ cells were placed in each egg as previously reported [30]. Day 4 versus day 7 cell placement was evaluated exclusively as part of condition A. Once we observed that day 7 resulted in improved outcomes, all subsequent experiments were performed using day 7 for tumor induction. After 6 days of incubation, a window of approximately 2 cm^2^ was opened in the blunt end of the egg. To expose the CAM, the eggshell membranes were removed with fine tweezers, and the eggshell window was sealed with transparent tape. The eggs were placed in a static incubator. After 7 days of incubation, cells with Matrigel were placed on the CAM and the eggshell window was sealed with transparent tape. The chorioallantoic membrane (CAM) was gently lacerated. This procedure was performed prior to tumor cell placement to induce a controlled superficial disruption of the CAM. A single gentle laceration was made using the tip of a sterile 200 μL pipette, sufficient to disrupt the epithelial layer and produce minimal localized bleeding confined to the inoculation area, visually confirmed within a diameter of approximately 1–2 mm. Following this procedure, the eggs were returned to a static incubator at 37 °C for an additional four days. The eggs were placed into a static incubator at 37 °C for another four days. The cell processing before placing them onto the CAM was as follows: HeLa and SiHa cells were counted in a hemocytometer using the trypan blue exclusion method. Subsequently, 3 × 10^6^ cells were resuspended in 20 μL of Matrigel and incubated for 20 min at 37 °C and 5% CO_2_. The suspension was prepared in a 1.5 mL tube.

The tube was introduced into the cell culture incubator for 20 min, and the cell suspension was incubated and placed onto the CAM.

### 2.3. Morphological Analysis and Histological Processing

Tumors were photographed when they were still on the CAM. After the macroscopic morphology exam, the tumors were fixed in neutral formalin. Then, the same tumors were dehydrated through increasing series in alcohol (70–100%), rinsed with xylene (Sigma-Aldrich, Burlington, MA, USA), and embedded in paraplast paraffin wax (Tissue-Tek, Torrance, CA, USA). The tissue was cut into 6 µm cross-sections and stained with hematoxylin and eosin (HE; Hycel, Zapopan, Jalisco, Mexico). Panoramic histological images were acquired using a Digital Aperio Scanner (Leica, Wetzlar, Germany) and the ImageScope software version 12.4.6.

### 2.4. Immunofluorescence

The levels of Ki-67 expression were estimated using immunofluorescence with primary antibodies (dilution 1:200) (sc-23900 Santa Cruz Biotechnology, Dallas, TX, USA). The samples were first incubated overnight at 4 °C with primary antibodies and then treated with fluorency-labeled anti-rabbit secondary antibodies for 4 h at room temperature. The cell nuclei were stained with 1:150 RedDot dilution (Biotium, Freemont, CA, USA). Finally, the samples were mounted and observed under a confocal microscope (Carl Zeiss, Oberkochen, Germany). Pictures of three random areas of the tumor were taken using ZEN 2010 software (Carl Zeiss, Germany). Optical density was reported as the mean ± standard deviation.

### 2.5. Statistical Analysis

The statistical analysis was conducted using Jamovi software version 2.6.45. The number of eggs where tumors were induced per group was recorded and expressed as relative frequency. Morphometric data, and Ki67 relative fluorescence intensity were expressed as mean ± SD. A Student’s *t*-test was applied to compare the groups, and a *p* < 0.05 was considered statistically significant.

## 3. Results

### 3.1. Important Conditions for In Ovo Tumor Induction

**Figure 1:**
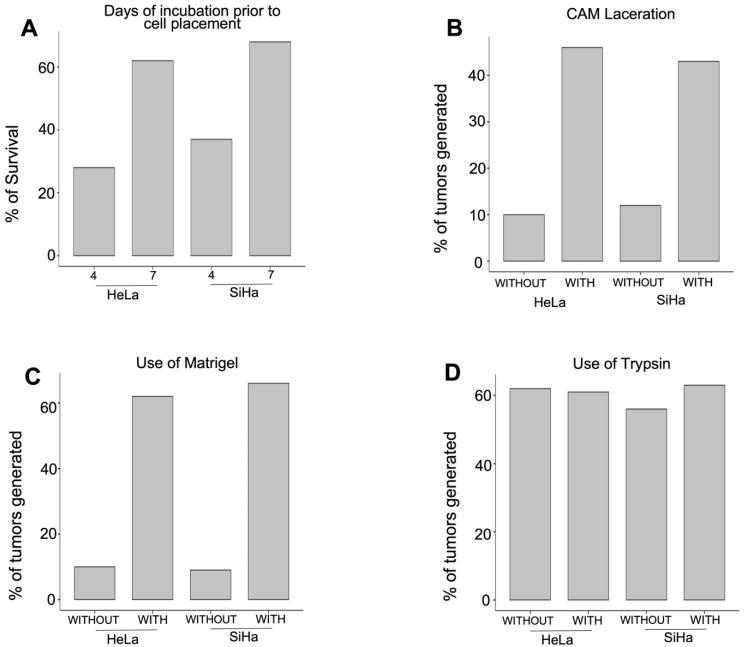
Conditions that Favor the Generation of Tumors. (**A**) The embryo survival improves when tumors are induced by day 7. (**B**) Lightly lacerating the CAM causes an increase in the number of tumors generated. (**C**) Besides lacerating the CAM, the number of tumors increases if cells are placed in Matrigel. (**D**) No difference was observed in tumor establishment whether or not trypsin was used.

The number of cells placed onto the CAM may differ among various research studies that explore this subject. However, in this study, 3 million cells from both cell lines were used, as previously reported by Martinho et al. (2013) [30]. The aim was to determine whether embryo survival improves when cells are applied during the later days of incubation. In this case, the cell placement was performed on day 7 of embryo incubation, which improves the survival rate of up to 62% and 68% for HeLa and SiHa cell lines, respectively. An attempt was also made to perform inoculation on day 4 of incubation, but the embryos at this stage are so small and fragile that removing the eggshell membranes can negatively impact their survival rate (Figure 1A). After determining the optimal day for cell application onto the CAM, we explored tumor establishment by either lacerating or leaving the CAM intact. Considering the protective role of this extraembryonic membrane, tumor establishment was more successful when the CAM was lacerated. We used a pipette tip (20–200 μL) to create a small injury in the CAM, resulting in minor bleeding. We found that tumors developed in 46% of the inoculated eggs with HeLa cells and 43% with SiHa cells when the CAM was lacerated and only 12% (HeLa) and 10% (SiHa) tumors developed when the CAM was intact (Figure 1B). Matrigel proved to be critical in our experiments for optimal tumor establishment. We attempted to place the cells without Matrigel and found that in a few cases, tumor generation occurred due to cell dispersion, creating a similar monolayer on the CAM. Adding a support material to the cell suspension prevents cell dispersion and reduces cell death due to anoikis, thus preserving cell numbers.

We attempted to enhance tumor formation by adding trypsin to the CAM; however, no significant difference was observed in using trypsin or not (Figure 1D).

### 3.2. Time of Tumor Formation in the CAM

**Figure 2:**
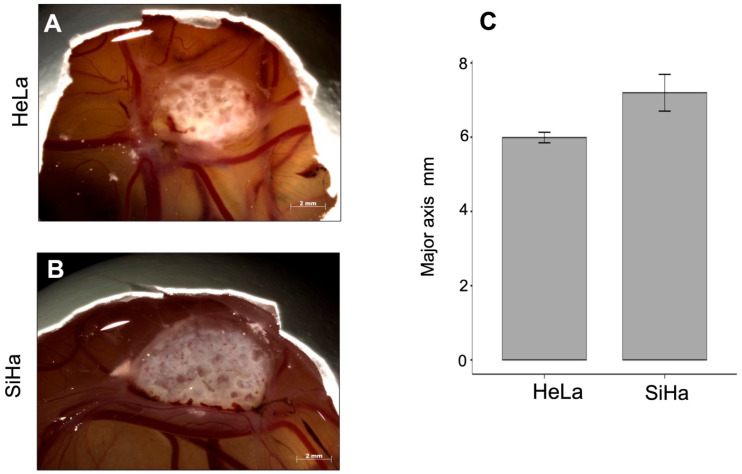
Generation of *in ovo* tumors. By the third or fourth day, after placing the cells, the appearance of both cell line tumors can be observed in the CAM. (**A**,**B**) The morphology of the tumors is observed *in ovo*, and these tumors are surrounded by blood vessels. (**C**) The major axis of the tumors was measured and SiHa generates bigger tumors in comparison with HeLa. Data are presented as mean ± SD (HeLa, 5.99 ± 0.25, n = 3; SiHa, 7.20 ± 0.86, n = 3). Statistical significance was assessed using Student’s *t*-test, and a *p*-value < 0.05 was considered statistically significant.

In the chick embryo CAM, tumor growth can be detected as early as three days post-inoculation. In addition to this advantage, as shown in Figure 2A,B, the tumors are exposed and can be easily manipulated and photographed. Tumor size, measured along the major axis, was 5.99 ± 0.25 mm in HeLa-derived tumors (n = 3) and 7.20 ± 0.86 mm in SiHa-derived tumors (n = 5), with no statistically significant difference between groups (*p* = 0.075; Figure 2C).

### 3.3. Tumors Formed in the CAM Display Vascular-like Structures

**Figure 3:**
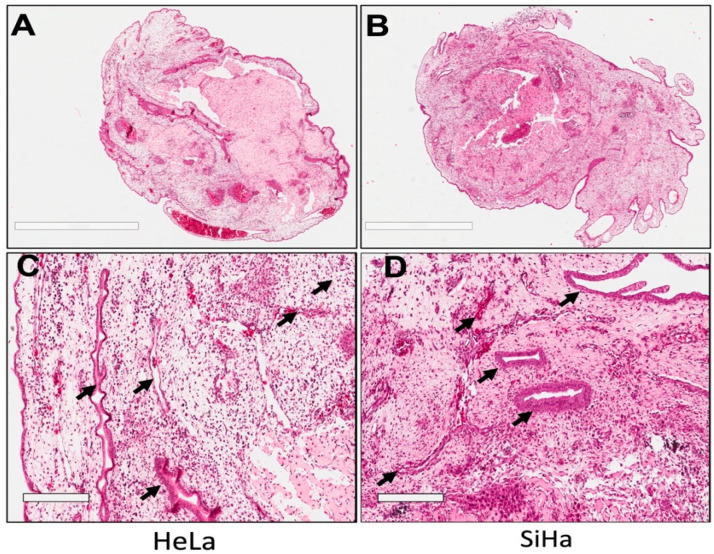
Histological analysis of the tumors induced on the CAM. The CAM environment provides the necessary conditions for both tumor growth and tumor vascularization. A complete tumor section from (**A**) HeLa and (**B**) SiHa is observed, where vascular-like structures are visible. Similarly, a 20× magnification of a tumor region within the (**C**) HeLa and (**D**) SiHa is displayed, where vascular-like structures are more clearly distinguished and even blood cells are visible within them (arrow indicated). All images were acquired at 40× magnification (scale bar = 2 mm in (**A**,**B**) and 200 μm in (**C**,**D**)).

One of the key characteristics of the CAM is its highly vascularized structure [28,31], which makes it a valuable model for studying tumor angiogenesis [32,33]. Histological evaluation using hematoxylin and eosin (H&E) staining revealed the presence of vascular-like structures associated with tumors formed in the CAM from cervical cancer cell lines. Figure 3 shows cross-sectional views of complete tumors derived from HeLa (Figure 3A) and SiHa (Figure 3B) cell implants. Higher magnification images (Figure 3C,D) illustrate lumen-like structures containing erythrocytes (indicated by arrows), which are consistent with perfused vascular-like structures within or surrounding the tumor mass. Tumors induced in the CAM are known to undergo an initial avascular phase during the first 3–4 days post-implantation [26]. Following this period, host-derived endothelial cells migrate toward the tumor mass, contributing to the formation of vascular-like structures that support tumor growth. In line with this established behavior of the CAM model, the histological findings presented here confirm that CAM-derived tumors develop vascular-associated features suitable for short-term tumor growth studies.

### 3.4. Ki-67 Expression in CAM-Derived Tumors

**Figure 4:**
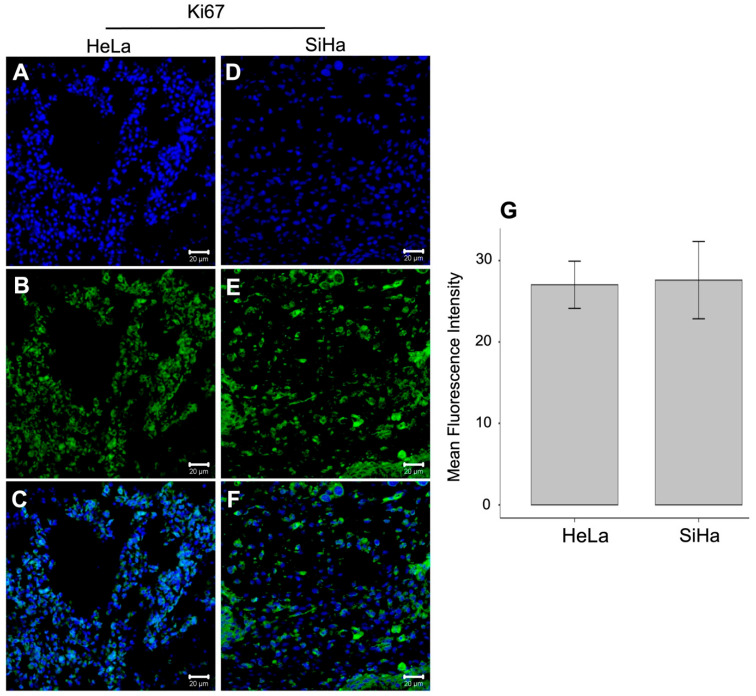
Analysis of Ki-67 expression *in ovo*-induced tumors. Ki-67 expression was evaluated by immunodetection in tumors derived from both cell lines. (**A**) The nuclear marker (RedDot) for tumors induced with the HeLa cell line, (**B**) Ki67 detection in tumors induced with HeLa, and (**C**) the merge are observed. It is observed likewise the (**D**) Ki67 nuclear marker from tumors induced with SiHa, the (**E**) Ki-67 nuclear marker in the tumors induced with SiHa and (**F**) the merge. (**G**) Mean Fluorescence Intensity, no statistically significant difference was observed in the Ki-67 expression of tumors from both cell lines. All images were acquired at 40× magnification. Scale bar = 2 μm. Data are presented as mean ± SD (HeLa, 27.0 ± 7.11, n = 6; SiHa, 27.6 ± 12.6, n = 7). Statistical significance was assessed using Student’s *t*-test, and a *p*-value < 0.05 was considered statistically significant. RedDot = Blue; Ki67 = Green.

**Figure 5:**
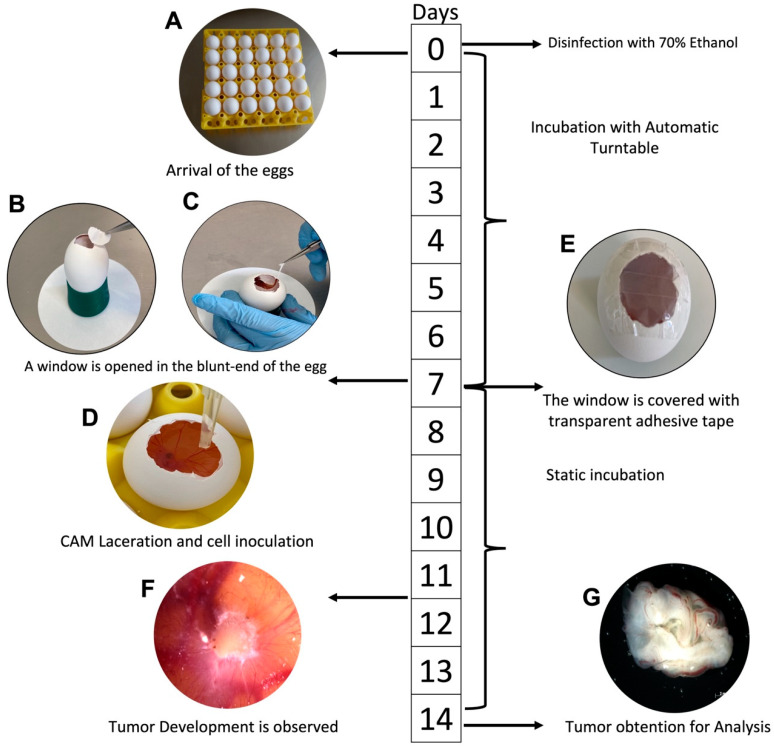
General Scheme of *in ovo* tumor induction using HeLa and SiHa cell lines. (**A**) The embryonated eggs are placed in a rotating incubator upon their arrival. (**B**,**C**) At 6 days of incubation, the CAM is exposed. (**D**) At 7 days of incubation, cells with Matrigel are placed into the CAM after laceration. (**E**) The window is covered with transparent adhesive tape and left in static incubation. (**F**) After day 10, the tumors are already established in the CAM and ready for experimentation. (**G**) In our research, the tumors were removed from the CAM on day 14.

Immunodetection of Ki-67 was performed in the tumors induced onto the CAM, and the relative fluorescence units were measured in tumors derived from both cell lines: The mean fluorescence intensity of Ki-67 was 27.0 ± 7.11 in HeLa-derived tumors (n = 6; Figure 4A–C) and 27.6 ± 12.6 in SiHa-derived tumors (n = 7; Figure 4D–F). No statistically significant difference in Ki-67 expression was observed between the two groups (*p* = 0.924). Our results indicate that the CAM model is a viable exploratory model for the research on tumors derived from CC. Figure 5 presents the procedures for tumor induction in the chicken chorioallantoic membrane based on HeLa and SiHa cell lines derived from CC.

## 4. Discussion

Tumor induction on the chicken chorioallantoic membrane (CAM) represents an efficient, rapid, and cost-effective in vivo platform for cancer research. Since its first application by Rous in 1911 to study chicken sarcoma growth [34], the CAM model has been extensively employed in embryology to investigate development, morphogenesis, and organogenesis [35], and later expanded to biomedical research, including cancer biology, angiogenesis, metastasis, and antineoplastic drug screening [24,25]. Despite its broad application across multiple tumor types, the use of the CAM assay for studying cervical cancer tumor biology remains relatively underexplored. From an ethical standpoint, the CAM model aligns well with the principles of Replacement, Reduction, and Refinement (3Rs), as experimentation is conducted before the onset of nociception and often does not require formal ethical committee approval in many jurisdictions [23,25]. These characteristics make the CAM assay a particularly accessible exploratory platform. Several studies have previously demonstrated the feasibility of inducing cervical cancer tumors on the CAM. For example, Martinho et al. induced HeLa-derived tumors on day 10 of incubation using a plastic ring and reported tumors with a mean diameter of 4335 ± 823 μm [30]. In contrast, our approach omitted the use of a physical ring and instead relied on direct cell inoculation combined with Matrigel at day 7 of incubation, achieving consistent tumor formation while simplifying the technical procedure. Similarly, Zhu et al. (2022) employed the CAM assay to study angiogenesis in HeLa- and SiHa-derived tumors; however, critical methodological details, such as the exact number of cells inoculated, were not reported, limiting reproducibility [36]. Our study addresses this gap by providing a detailed and standardized description of technical variables influencing tumor induction efficiency. One of the principal advantages of the CAM assay is the accelerated timeline of tumor formation. The tumors in the CAM model can be detected within three to four days post-implantation. Although tumor induction has been reported as early as days 3–4 of incubation [37], our results indicate that performing cell inoculation on day 7 improves embryo survival, likely due to increased resistance to manipulation at later developmental stages. Importantly, the chick embryo remains naturally immunosuppressed prior to day 10, facilitating xenograft engraftment with minimal immune rejection, as T and B lymphocytes appear only after days 11 and 12, respectively [23,38,39]. Minor laceration of the CAM has been reported to enhance tumor engraftment by facilitating cell invasion [37,40]. Consistent with these reports, we performed a controlled superficial laceration using a pipette tip. While trypsin treatment has been proposed as an additional strategy to promote implantation [41], our findings indicate that trypsin was not necessary for successful tumor formation using HeLa and SiHa cells. This observation may be explained by the intrinsic invasive capacity of cervical cancer cells, which express high levels of matrix metalloproteinases, including MMP-2, MMP-9, MMP-11, and MMP-12, all of which play critical roles in extracellular matrix degradation and tissue invasion [42,43]. Both HeLa and SiHa cells have been reported to secrete functional MMPs in vitro, supporting their efficient invasion of the CAM epithelial layer [44]. Robust vascularization was consistently observed in the induced tumors, reflecting the highly angiogenic microenvironment of the CAM. This structure is rich in pro-angiogenic factors such as VEGF, FGF-2, angiopoietin-1, and HIF-1α, which reach peak expression between days 11 and 12 of incubation [45,46,47]. While our study did not directly evaluate antiangiogenic therapies, the observed vessel-like structures support the utility of the CAM assay for studying tumor-associated angiogenesis in cervical cancer. The vessel-like structures and Ki67 expression observed in CAM-induced cervical cancer tumors may reflect key hallmarks of human cervical carcinoma in vivo. In patients, cervical tumors are characterized by dense and disorganized vascular networks driven by high expression of pro-angiogenic factors such as VEGF and HIF-1α, which are associated with tumor progression and therapeutic response [42,43]. Although the CAM vasculature does not fully recapitulate the structural complexity of human tumor-associated vessels, its rapid neovascularization and permissive microenvironment allow the assessment of angiogenic patterns and tumor–stroma interactions at early stages [24,37]. Similarly, the Ki67 signal detected in CAM-induced tumors is consistent with the reported findings in human cervical cancer, where markers such as Ki67 correlate with tumor aggressiveness [48,49]. While molecular and immune microenvironmental components are inherently simplified in the CAM model, these shared features support its utility for early-stage biological studies and preliminary drug screening, particularly for agents targeting proliferation or angiogenesis, prior to validation in more complex mammalian systems. Despite these advantages, the CAM model presents inherent biological limitations that must be acknowledged. The restricted experimental window, typically ending around day 14 of embryonic development, limits long-term studies of tumor progression, metastasis, and delayed therapeutic responses [37,50,51]. Additionally, the immunologically immature environment of the chick embryo does not recapitulate the complexity of tumor–immune interactions present in adult organisms, which constrains its applicability for immunotherapy studies [52,53]. Consequently, the CAM assay should be regarded as a complementary preclinical model rather than a replacement for established murine systems. The need for replacing experimental animals with models with less capacity for feeling pain or discomfort, or simply reducing the number of animals used in experimentation, remains a great challenge in the research field. The chorioallantoic membrane not only addresses this challenge but also represents a simple, low-cost, short-term exploratory model for studying the different aspects of cervical cancer development biology such as tumorigenicity, angiogenesis, metastasis, or even for proving the effects of antineoplastic drugs. Quantitative analyses were performed on only a subset of tumors. While this approach ensured reliable measurements, it may limit the generalizability of the quantitative findings. Accordingly, the results should be interpreted as descriptive within the context of this exploratory CAM-based model.

## 5. Conclusions

We have described an effective and rapid method for tumor induction through xenotransplantation of CC-derived cell lines (HeLa and SiHa) using the chick embryo chorioallantoic membrane assay. Tumors generated on the CAM exhibited organized tissue architecture and the presence of vessel-like structures. In addition, Ki-67 expression was detected in CAM-derived tumors based on relative fluorescence intensity. Due to its ease of use, accessibility, and low cost, this approach represents a practical short-term exploratory in vivo model for the study of CC-derived tumors.

## Data Availability

The original contributions presented in this study are included in the article. Further inquiries can be directed to the corresponding author.

## References

[1] Cubie H.A. (2013). Diseases associated with human papillomavirus infection. Virology.

[2] Cunha A.P.A., Belfort I.K.P., Mendes F.P.B., Dos Santos G.R.B., Costa L.H.L., de Matos Monteiro P., Gaspar R.L., Ferreira M.B., de Sá Ferreira A., Monteiro S.C.M. (2020). Human papillomavirus and its association with other sexually transmitted coinfection among sexually active women from the Northeast of Brazil. Interdiscip. Perspect. Infect. Dis..

[3] Rahangdale L., Mungo C., O’Connor S., Chibwesha C.J., Brewer N.T. (2022). Human papillomavirus vaccination and cervical cancer risk. BMJ.

[4] Speer L., Bodi S. (2021). Cervical cancer update: The latest on screening & management. J. Fam. Pract..

[5] Mitra T., Elangovan S. (2021). Cervical cancer development, chemoresistance, and therapy: A snapshot of involvement of microRNA. Mol. Cell. Biochem..

[6] Bhattacharjee R., Dey T., Kumar L., Kar S., Sarkar R., Ghorai M., Malik S., Jha N.K., Vellingiri B., Kesari K.K. (2022). Cellular landscaping of cisplatin resistance in cervical cancer. Biomed. Pharmacother..

[7] Bongso A., Gajra B., Lian N.P., Wong P.C., Soon-Chye N., Ratnam S. (1988). Establishment of human endometrial cell cultures. Hum. Reprod..

[8] Hirte H.W., Clark D.A., Mazurka J., O’Connell G., Rusthoven J. (1992). A rapid and simple method for the purification of tumor cells from ascitic fluid of ovarian carcinoma. Gynecol. Oncol..

[9] Karst A.M., Drapkin R. (2012). Primary culture and immortalization of human fallopian tube secretory epithelial cells. Nat. Protoc..

[10] Shepherd T.G., Thériault B.L., Campbell E.J., Nachtigal M.W. (2006). Primary culture of ovarian surface epithelial cells and ascites-derived ovarian cancer cells from patients. Nat. Protoc..

[11] Stanley M.A., Parkinson E.K. (1979). Growth requirements of human cervical epithelial cells in culture. Int. J. Cancer.

[12] Greely H.T., Cho M.K. (2013). The Henrietta Lacks legacy grows. EMBO Rep..

[13] Horbach S.P.J.M., Halffman W. (2017). The ghosts of HeLa: How cell line misidentification contaminates the scientific literature. PLoS ONE.

[14] Skok K., Gradišnik L., Maver U., Kozar N., Sobočan M., Takač I., Arko D., Kavalar R. (2021). Gynaecological cancers and their cell lines. J. Cell. Mol. Med..

[15] Kutle I., Polten R., Hachenberg J., Klapdor R., Morgan M., Schambach A. (2023). Tumor organoid and spheroid models for cervical cancer. Cancers.

[16] Lõhmussaar K., Oka R., Espejo Valle-Inclan J., Smits M.H.H., Wardak H., Korving J., Begthel H., Proost N., van de Ven M., Kranenburg O.W. (2021). Patient-derived organoids model cervical tissue dynamics and viral oncogenesis in cervical cancer. Cell Stem Cell.

[17] Wang Z., Lv J., Zhang T. (2015). Combination of IL-24 and cisplatin inhibits angiogenesis and lymphangiogenesis of cervical cancer xenografts in a nude mouse model by inhibiting VEGF, VEGF-C and PDGF-B. Oncol. Rep..

[18] Wei W.F., Han L.F., Liu D., Wu L.F., Chen X.J., Yi H.Y., Wu X.G., Zhong M., Yu Y.H., Liang L. (2017). Orthotopic Xenograft Mouse Model of Cervical Cancer for Studying the Role of MicroRNA-21 in Promoting Lymph Node Metastasis. Int. J. Gynecol. Cancer.

[19] Bulut G., Üren A. (2015). Generation of K14-E7/∆N87βcat Double Transgenic Mice as a Model of Cervical Cancer. Methods Mol. Biol..

[20] Li C., Zhang L., Liu C., He X., Chen M., Chen J. (2022). Lipophilic Grape Seed Proanthocyanidin Exerts Anti-Cervical Cancer Effects in HeLa Cells and a HeLa-Derived Xenograft Zebrafish Model. Antioxidants.

[21] Brown H.K., Schiavone K., Tazzyman S., Heymann D., Chico T.J. (2017). Zebrafish Xenograft Models of Cancer and Metastasis for Drug Discovery. Expert Opin. Drug Discov..

[22] Zhang B., Xuan C., Ji Y., Zhang W., Wang D. (2015). Zebrafish Xenotransplantation as a Tool for In Vivo Cancer Study. Fam. Cancer.

[23] Kundeková B., Máčajová M., Meta M., Čavarga I., Bilčík B. (2021). Chorioallantoic Membrane Models of Various Avian Species: Differences and Applications. Biology.

[24] Ribatti D. (2014). The Chick Embryo Chorioallantoic Membrane as a Model for Tumor Biology. Exp. Cell Res..

[25] Ribatti D. (2016). The Chick Embryo Chorioallantoic Membrane (CAM). A Multifaceted Experimental Model. Mech. Devices.

[26] Knighton D., Ausprunk D., Tapper D., Folkman J. (1977). Avascular and Vascular Phases of Tumour Growth in the Chick Embryo. Br. J. Cancer.

[27] Komatsu A., Matsumoto K., Yoshimatsu Y., Okada Y., Miyagawa Y., Orita H., Fukushi J., Iwamoto Y., Matsunobu T. (2021). The CAM model for CIC-DUX4 sarcoma and its potential use for precision medicine. Cells.

[28] Ribatti D. (2021). The CAM Assay in the Study of the Metastatic Process. Exp. Cell Res..

[29] Ribatti D. (2008). Chick Embryo Chorioallantoic Membrane as a Useful Tool to Study Angiogenesis. Int. Rev. Cell Mol. Biol..

[30] Martinho O., Pinto F., Granja S., Miranda-Gonçalves V., Moreira M.A., Ribeiro L.F., Monteiro A.R., Carvalho A.S., Reis R.M. (2013). RKIP inhibition in cervical cancer is associated with higher tumor aggressive behavior and resistance to cisplatin therapy. PLoS ONE.

[31] Deryugina E.I., Quigley J.P. (2008). Chick embryo chorioallantoic membrane models to quantify angiogenesis induced by inflammatory and tumor cells or purified effector molecules. Methods Enzymol..

[32] Ribatti D. (2008). The Chick Embryo Chorioallantoic Membrane in the Study of Tumor Angiogenesis. Rom. J. Morphol. Embryol..

[33] Hasan J., Shnyder S.D., Bibby M., Double J.A., Bicknel R., Jayson G.C. (2004). Quantitative angiogenesis assays in vivo—A review. Angiogenesis.

[34] Rous P., Murphy J.B. (1911). Tumor implantations in the developing embryo. J. Am. Med. Assoc..

[35] Hamburger V., Hamilton H.L. (1951). A series of normal stages in the development of the chick embryo. J. Morphol..

[36] Zhu K., Deng C., Du P., Liu T., Piao J., Piao Y., Yang M., Chen L. (2022). G6PC indicated poor prognosis in cervical cancer and promoted cervical carcinogenesis in vitro and in vivo. Reprod. Biol. Endocrinol..

[37] Nowak-Sliwinska P., Segura T., Iruela-Arispe M.L. (2014). The chicken chorioallantoic membrane model in biology, medicine and bioengineering. Angiogenesis.

[38] Janković B.D., Isaković K., Lukić M.L., Vujanović N.L., Petrović S., Marković B.M. (1975). Immunological capacity of the chicken embryo. I. Relationship between the maturation of lymphoid tissues and the occurrence of cell-mediated immunity in the developing chicken embryo. Immunology.

[39] Le Douarin N.M., Jotereau F.V. (1975). Tracing of cells of the avian thymus through embryonic life in interspecific chimeras. J. Exp. Med..

[40] Ribatti D., Annese T. (2023). Chick embryo in experimental embryology and more. Pathol. Res. Pract..

[41] Zijlstra A., Aimes R.T., Zhu D., Regazzoni K., Kupriyanova T., Seandel M., Quigley J.P. (2004). Collagenolysis-dependent angiogenesis mediated by matrix metalloproteinase-13 (collagenase-3). J. Biol. Chem..

[42] Rauvala M., Aglund K., Puistola U., Turpeenniemi-Hujanen T., Horvath G., Willén R., Stendahl U. (2006). Matrix metalloproteinases-2 and -9 in cervical cancer: Different roles in tumor progression. Int. J. Gynecol. Cancer.

[43] Davidson B., Goldberg I., Gotlieb W.H., Kopolovic J., Ben-Baruch G., Nesland J.M., Reich R. (2002). The prognostic value of metalloproteinases and angiogenic factors in ovarian carcinoma. Mol. Cell. Endocrinol..

[44] Roomi M.W., Monterrey J.C., Kalinovsky T., Rath M., Niedzwiecki A. (2010). In vitro modulation of MMP-2 and MMP-9 in human cervical and ovarian cancer cell lines by cytokines, inducers and inhibitors. Oncol. Rep..

[45] Baum O., Suter F., Gerber B., Tschanz S.A., Buergy R., Blank F., Hlushchuk R., Djonov V. (2010). VEGF-A promotes intussusceptive angiogenesis in the developing chicken chorioallantoic membrane. Microcirculation.

[46] Makanya A.N., Hlushchuk R., Djonov V. (2009). Intussusceptive angiogenesis and its role in vascular morphogenesis, patterning, and remodeling. Angiogenesis.

[47] Ribatti D., Tamma R., Annese T. (2021). Chorioallantoic membrane vascularization. A meta-analysis. Exp. Cell Res..

[48] Pinto A.P., Crum C.P. (2000). Natural history of cervical neoplasia: Defining progression and its consequence. Clin. Obstet. Gynecol..

[49] Pan D., Wei K., Ling Y., Su S., Zhu M., Chen G. (2015). The prognostic role of Ki-67/MIB-1 in cervical cancer: A systematic review with meta-analysis. Med. Sci. Monit..

[50] Chu P.Y., Koh A.P.F., Antony J., Huang R.Y.J. (2022). Applications of the chick chorioallantoic membrane as an alternative model for cancer studies. Cells Tissues Organs.

[51] Patiño-Morales C.C., Jaime-Cruz R., Ramírez-Fuentes T.C., Villavicencio-Guzmán L., Salazar-García M. (2023). Technical implications of the chicken embryo chorioallantoic membrane assay to elucidate neuroblastoma biology. Int. J. Mol. Sci..

[52] Miebach L., Berner J., Bekeschus S. (2022). In ovo model in cancer research and tumor immunology. Front. Immunol..

[53] Ribatti D. (2017). The chick embryo chorioallantoic membrane (CAM) assay. Reprod. Toxicol..

